# Toward confirmation of the safety and efficacy of methoxy polyethylene glycol-epoetin beta in anemia treatment in patients on hemodialysis: a Macedonian experience

**DOI:** 10.3325/cmj.2019.60.475

**Published:** 2019-10

**Authors:** Isidora Kacarska-Fotevska, Nadja Volckova, Katerina Ilievska, Doncho Donev

**Affiliations:** 1 Hoffmann La Roche Ltd., Representative Office, Skopje, North Macedonia; 2 Roche Macedonia DOOEL, Skopje, North Macedonia; 3 Institute of Social Medicine, Faculty of Medicine, Skopje, North Macedonia *dmdonev@gmail.com*

Anemia is a common complication in patients with chronic kidney disease (CKD). The primary cause of anemia, present in about 50% of pre-dialysis patients and over 90% of hemodialysis patients, is endogenous erythropoietin deficiency. If left untreated, chronic anemia may affect patients’ quality of life (QoL), leading to significant morbidity and mortality. Despite the use of erythropoiesis-stimulating agent (ESA) as a standard therapy for renal anemia, anemia management in CKD remains a challenge from the treatment point of view (1-5).

The main impact of anemia is reduced oxygen delivery to tissues, leading to fatigue and dyspnea, reduced exercise tolerance, and poor health-related QoL. Other consequences, such as impaired cognitive function, sleep disorders and depression, altered hemostasis, depressed immune function, and impaired cardiac function, are not uncommon (1,6,7).

Patients with CKD need additional ESA treatment. А number of articles discussed the benefits and possible risks associated with ESA treatment (2,8-10). Many clinical trials and observational studies in CKD populations, both on and off dialysis, demonstrated the benefits of normalizing hemoglobin (Hb) levels. This prompted frequent revisions of the guidelines for the management of CKD anemia (5,7,11-16).

The key question addressed was whether the optimal Hb target for CKD patients in the range of 110.0 to 120.0 g/L was achieved. Challenges to the effectiveness and safety of ESAs were emphasized when some clinical trials showed no benefit or induced harm related to cardiovascular outcomes in patients targeted to higher Hb levels. In CKD patients receiving ESA therapy, the Hb target should not be lower than 90.0 g/L and greater than 130.0 g/L. It is reasonable to personalize therapy as some patients may experience improvements in QoL at higher Hb concentration. It means that ESA therapy may start above 100.0 g/L. The correction of Hb concentration should be based on post dialysis values, namely dry Hb concentrations (17-21).

Many guidelines provide advice to physicians on how to screen CKD patients for anemia and when and how to treat patients with different medications. It is essential to know how to safely prescribe epoetin ESAs and route of administration, as well as assess and optimize iron stores. In addition, it is important to know how to diagnose and manage complications associated with anemia and the drugs administered for its treatment. Guidelines also help define the areas where evidence is lacking and research is needed (6,17,18,22-24).

Exogenous replacement of erythropoietin by recombinant human erythropoietin as ESAs has become a widely accepted therapy of renal anemia. Three forms are available for clinical use: epoetin alpha, epoetin beta (darbepoetin alpha), and methoxy polyethylene glycol-epoetin beta as continuous erythropoietin receptor activator (C.E.R.A. or Mircera®). The treatment of patients on hemodialysis with ESAs has greatly advanced the management of renal anemia (8-10,25).

Epoetin alpha has relatively short half-life of 8.5 hours and requires more frequent administration, generally two to three times per week. Darbepoetin alpha has three times longer half-life (25 hours), which allows less frequent dosing to treat anemia in CKD. It allows higher patient compliance and convenience and minimizes the burden on staff caused by frequent administration. Darbepoetin alpha is as effective as epoetin alpha for maintaining Hb concentration when administered intravenously (i.v.) or subcutaneously (s.c.) once weekly or once every other week (9,10).

However, darbepoetin alpha therapy was often associated with recurrent cyclic fluctuations in Hb levels. Some studies showed that more than 90% of patients experienced Hb cycling with the amplitude 25.1 ± 9 g/L and duration of Hb excursions more than 8 weeks. ESA therapy should be tailored to the patient’s profile by using the smallest possible dose to control anemia symptoms and achieve Hb target level. It should also be accepted that Hb excursions above and below the target will occur from time to time (14,21,26).

C.E.R.A. is an ESA with a long half-life (approximately 130 hours) that allows longer (once monthly) dosing intervals for anemia treatment in CKD patients on and off dialysis. The recommended initial drug dose for patients not receiving ESA is 6 µg/kg body weight administered as a single i.v. or s.c. injection once every two weeks. Once Hb has been maintained between 100-120 g/L, C.E.R.A. may be administered once monthly using a double dose of 12 µg/kg. C.E.R.A. dosage is based on the total weekly ESA dose at the time of conversion ranging from once monthly dose of 120-360 µg to 60-180 µg once every two weeks. When C.E.R.A. therapy is initiated or adjusted, Hb should be monitored every two weeks until stabilized, and every two to four weeks thereafter (13,25,27,28).

Patients with CKD on dialysis who had previously been treated with an ESA maintained stable Hb levels (within ±10 g/L of baseline and within the range of 100-130 g/L) when directly converted to C.E.R.A. administered i.v. or s.c. every 2 or 4 weeks tailored to the clinical response. C.E.R.A. has been administered to more than 2000 CKD patients in clinical studies to date, providing a firm understanding of its efficacy, safety, and tolerability (7,13-16,27-32).

The European Medicines Agency (EMEA) Committee for Human Medicinal Products (CHMP) concluded that the benefits of ESAs products continued to outweigh their risks in the approved indications if used for maintaining the target Hb range of 100-120 g/L. The EMEA-CHMP issued a Public Statement to physicians to use ESAs strictly in accordance with their approved Summary of Product Characteristics regarding the indications and dosing recommendations. The European Renal Best Practice Guidelines recommend a Hb range 110-120 g/L without intentionally exceeding 130 g/L. In addition, the EMEA-CHMP emphasized the need to increase the scientific knowledge on the effects of ESAs and expressed the readiness to continue to review the safety profile of epoetins within the terms of their currently authorized indications in the EU as additional data becomes available (19,20).

Our study (ClinicalTrials.gov Registration No. NCT01422824 and Study ID Number ML25067) aimed to investigate and evaluate the safety, tolerability, and therapeutic efficacy of C.E.R.A. with once-monthly i.v. administration in hemodialysis patients with anemia previously treated with intravenous epoetin alfa or beta. We also assessed the adverse events (AEs) and maintenance of Hb concentrations. Тhis study expanded the volume and diversity of clinical safety experience and therapeutic efficacy of C.E.R.A. in order to inform future clinical practice and to demonstrate the safety and efficacy of C.E.R.A. in the context of the EMEA guidelines (20,32).

Among 185 patients enrolled in this study, in 184 we analyzed the safety and efficacy of C.E.R.A. with mean dose of 120.5 μg at the beginning of the study, and overall mean dose of 115.2 μg, with average 4.99 dose modifications per patient.

The preliminary results of our study confirm the known safety profile and tolerability, as well as the efficacy of C.E.R.A. in anemia management in CKD patients on dialysis. We identified 121 AEs in 49/184 patients. C.E.R.A. was generally well tolerated, with most AEs being of mild to moderate severity. In most cases the AEs were associated with co-morbidities in this group of patients. Mean Hb levels during the study varied but were always maintained stable within the target range of 100-120g/L. In addition, C.E.R.A. was convenient for a simplified regimen of anemia management with monthly dosing schedule compared with traditional frequently administered ESAs.
